# Recent advances in smart nanoplatforms for tumor non-interventional embolization therapy

**DOI:** 10.1186/s12951-022-01548-w

**Published:** 2022-07-20

**Authors:** Heng Dong, Dongliang Yang, Yanling Hu, Xuejiao Song

**Affiliations:** 1 Nanjing Stomatological Hospital, Medical School of Nanjing University Jiangsu, 30 Zhongyang Road, 210008 Nanjing, China; 2 School of Physical and Mathematical Sciences, Nanjing Tech University (NanjingTech), 30 South Puzhu Road, 211816 Nanjing, China; 3 Nanjing Polytechnic Institute, 210048 Nanjing, China

**Keywords:** Tumor non-interventional embolization therapy, Smart nanoplatforms, thrombus, Tumor vascular occlusion, Combination therapy

## Abstract

Tumor embolization therapy has attracted great attention due to its high efficiency in inhibiting tumor growth by cutting off tumor nutrition and oxygen supply by the embolic agent. Although transcatheter arterial embolization (TAE) is the mainstream technique in the clinic, there are still some limitations to be considered, especially the existence of high risks and complications. Recently, nanomaterials have drawn wide attention in disease diagnosis, drug delivery, and new types of therapies, such as photothermal therapy and photodynamic therapy, owing to their unique optical, thermal, convertible and in vivo transport properties. Furthermore, the utilization of nanoplatforms in tumor non-interventional embolization therapy has attracted the attention of researchers. Herein, the recent advances in this area are summarized in this review, which revealed three different types of nanoparticle strategies: (1) nanoparticles with active targeting effects or stimuli responsiveness (ultrasound and photothermal) for the safe delivery and responsive release of thrombin; (2) tumor microenvironment (copper and phosphate, acidity and GSH/H_2_O_2_)-responsive nanoparticles for embolization therapy with high specificity; and (3) peptide-based nanoparticles with mimic functions and excellent biocompatibility for tumor embolization therapy. The benefits and limitations of each kind of nanoparticle in tumor non-interventional embolization therapy will be highlighted. Investigations of nanoplatforms are undoubtedly of great significance, and some advanced nanoplatform systems have arrived at a new height and show potential applications in practical applications.

## Introduction

In tumor tissues, aggressive growth of tumor cells and overexpression of related proangiogenic factors lead to the development of disordered vascular networks. Compared with normal vessels, the complex tumor vasculature lacks a hierarchy, resulting in anomalies such as inconsistent vessel diameters, uneven shapes, and arteriovenous shunts [1, 2]. Such abnormal characteristics of the tumor vasculature would lead to typical microenvironmental conditions that hinder traditional antitumor therapeutic strategies such as chemotherapy and radiotherapy. For instance, impaired blood supply and interstitial hypertension inhibit drug delivery in solid tumors [3, 4]. On the other hand, the hypoxia induced by disordered vascular networks results in the resistance of tumor cells to clinical radiation therapy and antitumor drugs. In addition, hypoxia induces genetic instability and leads to increased metastasis of malignant cells [5–7]. Although the abnormal vasculature and the resulting abnormal microenvironment make the tumors more difficult to conquer, the unique features of the tumor vasculature provide opportunities for selective intervention [8–11].

The formation of new blood vessels not only meets the material requirements of tumor tissue and promotes tumor development but also provides a prerequisite for tumor cell invasion and metastasis. As early as 1971, Dr. Folkman proposed that the growth of tumors is significantly dependent on the blood supply. If there is no blood supply of nutrients and oxygen, tumors can only be in a dormant stage (1 ~ 2 mm in diameter) and subside with time [12]. Based on this theory, it is feasible to develop strategies to inhibit tumor growth by cutting off tumor nutrition and oxygen supply by an embolic agent [13, 14]. Theoretically, tumor embolization is very attractive for several reasons: (1) embolization of tumor vasculature can lead to the collapse of entire tumor vasculature networks; (2) the metastasis of solid tumors depends on the blood supply of nutrients and oxygen; therefore, tumor vascular embolization has great universality in different types of solid tumors; (3) since each tumor blood vessel is responsive to hundreds of tumor cells, tumor cell death could be efficiently induced in a short time during the process of embolization therapy; and (4) it can effectively reduce the risk of acquired drug resistance of tumor cells.

Transcatheter arterial embolization (TAE) is currently the mainstream technique for the clinical treatment of hypervascular and inoperable tumors [13, 15]. In the process of TAE, embolization agent is injected through a microcatheter to effectively block tumor blood arteries, thereby cutting off the supply of nutrition and oxygen for tumor growth and inhibiting tumor growth [16]. Although TAE is one of the most effective methods for treating middle-advanced tumors that cannot be surgically removed, there are still some limitations, such as the relatively complex and rigorous operation process, the relatively small scope of adaptation, and the existence of high risks and complications [17, 18]. Therefore, it is of great significance to develop novel non-invasive embolic agents with good tumor targeting, especially tumor vessel targeting, low toxicity and side effects and high performance, to achieve tumor non-interventional vascular embolization.

**Scheme 1:**
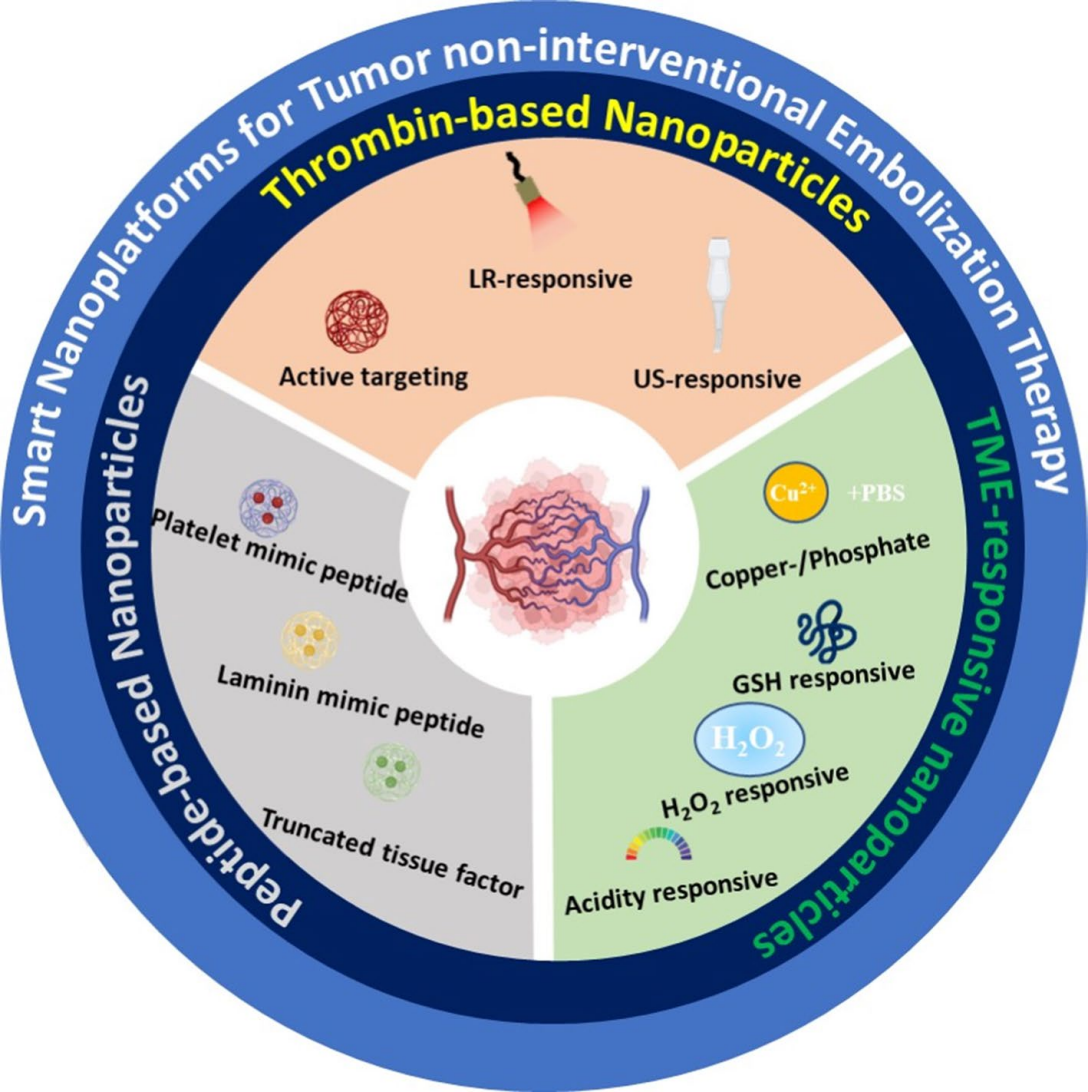
Smart nanoplatforms for tumor non-interventional embolization therapy

In tumor embolization therapy, the embolic material determines the effect of embolization. The embolization material used in embolization therapy should have the following characteristics: (1) no toxicity or side effects; (2) no rejection reaction or good biocompatibility; (3) easy visualization of the operation; and (4) long embolization time and no reopening phenomenon. The development of novel embolic materials is crucial for the further development of embolization therapy. In recent years, with the vigorous development of nanotechnology, nanomaterials have shown great potential in the field of biomedicine due to their unique optical, thermal, magnetic and in vivo transport properties, and they have provided more efficient and safer strategies for tumor treatments [19–21]. Nanotechnology offers unprecedented potential for tumor vascular embolization since researchers have used nanotechnology to safely deliver agents with coagulation activity or design responsive nanoplatforms to achieve highly specific embolization of tumor blood vessels. In this review, we summarize the nanoplatforms currently used for tumor non-interventional vascular embolization, mainly including different nanocarriers with the properties of high loading, safe delivery and responsive release of procoagulant substances for tumor non-interventional embolization therapy; different stimuli-responsive nanoparticles for highly specific tumor embolization therapy; and mimic nanomaterials based on peptides for precise embolization (Scheme 1). We will start from the embolization mechanism of various nanoplatforms and explore their important roles in inducing tumor embolization therapy and reducing toxic side effects. In addition, we will further discuss the application prospects and challenges of nanoplatforms in clinical tumor embolization therapy.

## Thrombin-based nanoparticles for tumor embolization therapy

The blood clot formed by the coagulation of the flowing blood in the vascular cavity or the cardiac cavity is called a thrombus, which can block the vascular cavity, significantly reduce the blood flow, cause severe tissue ischemia, and result in serious diseases [22, 23]. The formation of thrombi is very unfavorable to normal tissues, but thrombi can be turned from waste to treasure in tumor embolization therapy. It is a promising antitumor strategy to selectively promote tumor vascular thrombosis to induce tumor infarction necrosis.

**Fig. 1:**
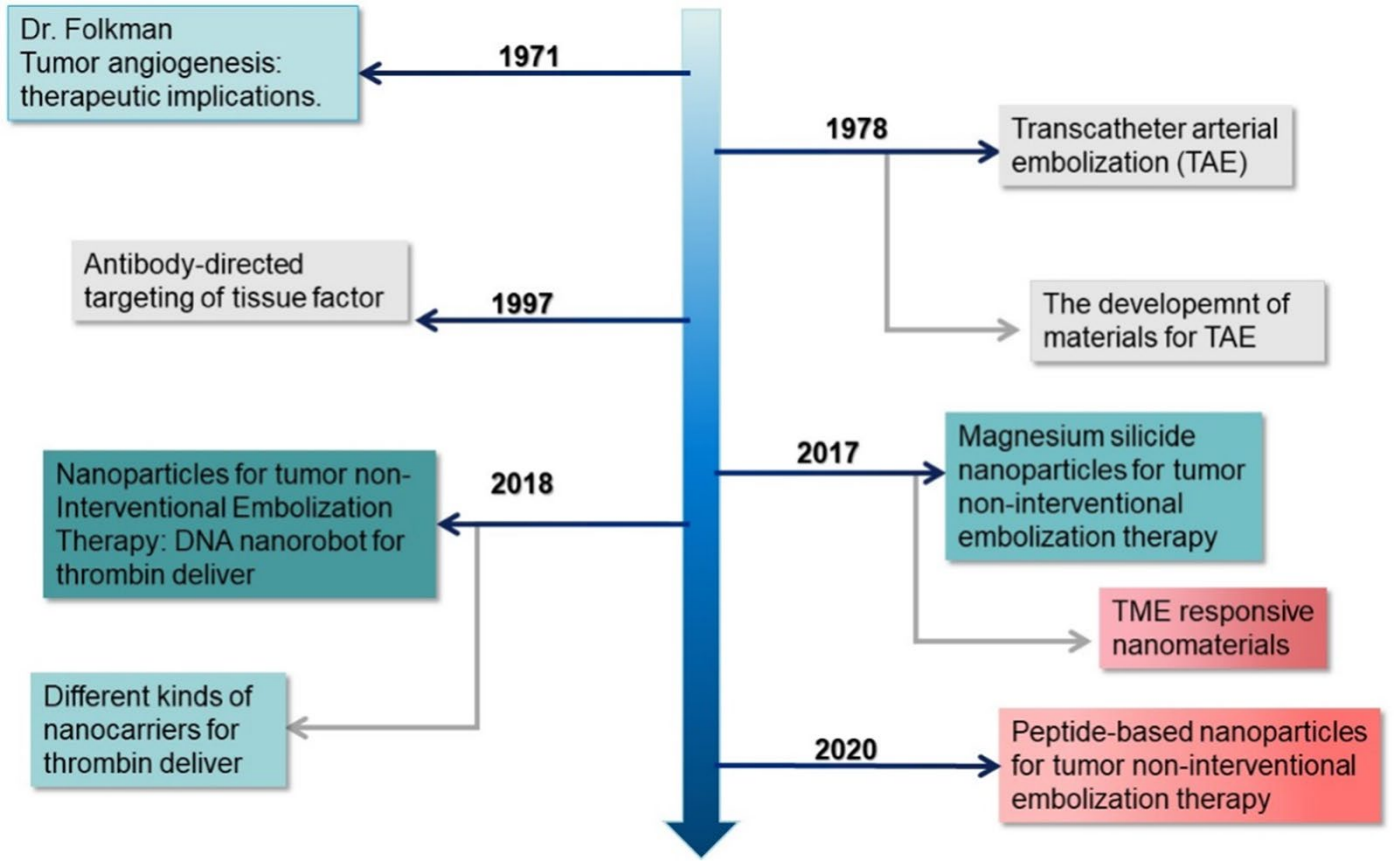
Evolution of different kinds of nanoparticles for tumor embolization in the past several decades

Thrombin has a strong coagulation function and can induce a coagulation reaction *via* efficiently activating platelets and converting fibrinogen into fibrin, thus resulting in local thrombi to exert hemostasis [24, 25]. These procoagulant substances can theoretically be used in tumor embolization therapy. Although tumor embolization has been developed for many years, the application of thrombin in tumor embolization did not appear until 2018 (Fig. 1). The main reason is that nonspecific thrombi are generated once thrombin contacts the blood, causing serious toxicity and side effects in tumor therapy. Therefore, constructing a carrier that can effectively encapsulate procoagulant substances, efficiently deliver them to target the local tumor, and achieve rapid drug release is an effective strategy to promote the further development of thrombin in tumor non-interventional vascular embolization therapy.

### Active targeting nanoparticles

**Fig. 2:**
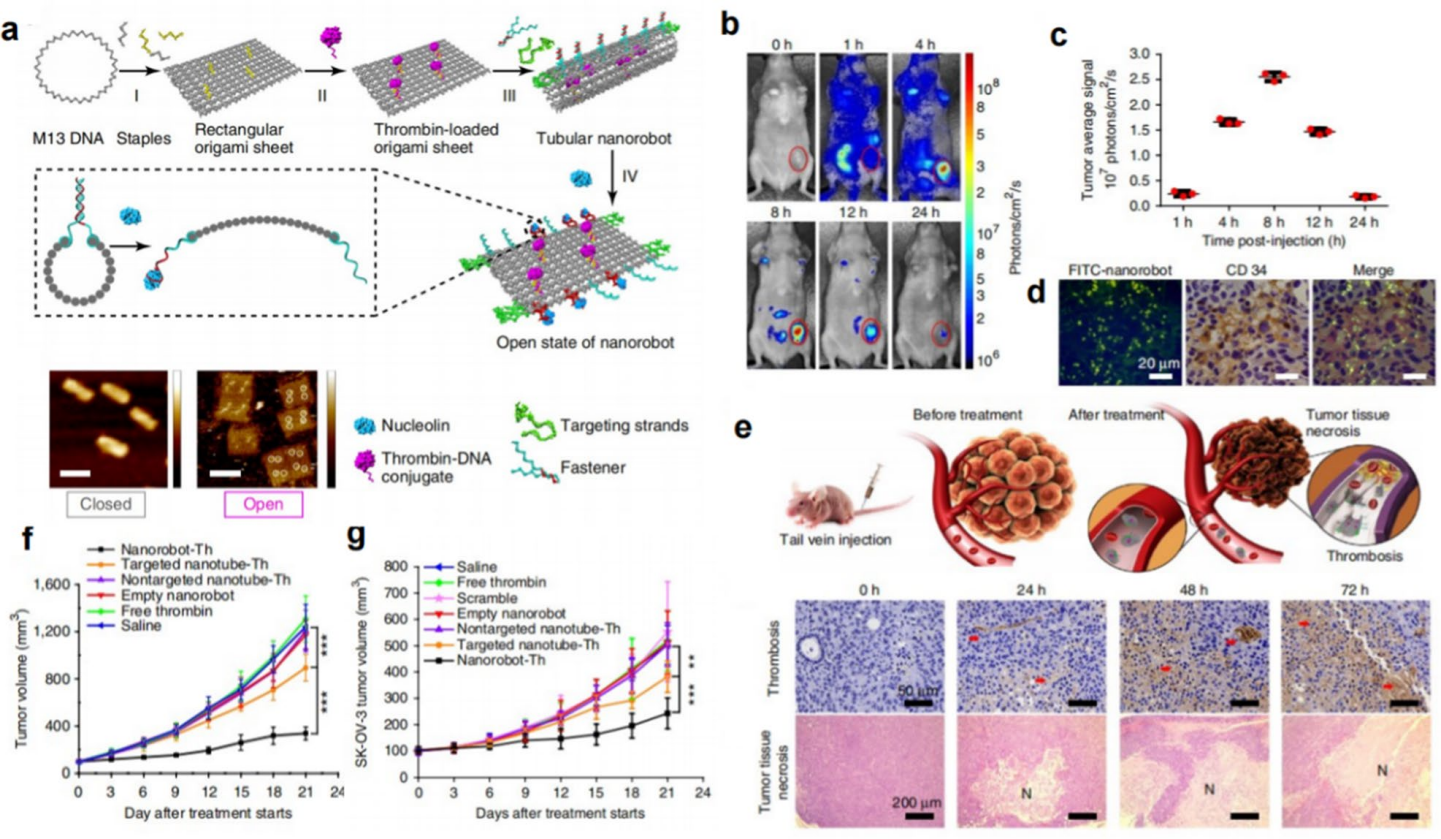
DNA nanorobot for the precise delivery of thrombin. **a** The design of a thrombin-loaded nanorobot by DNA origami. **b**, **c** Tumor accumulation of Cy5.5-labeled nanorobots in MDA-MB-231 mice bearing a human breast tumor at different time points after intravenous injection. **d** The active targeting effect of the aptamer-conjugated nanorobots on the tumor vascular endothelium. **e** The detection of the formation of thrombosis in the tumor and thrombosis-induced necrotic tissues was investigated by H&E staining assays. **f**, **g** The tumor growth inhibition effect with nanorobot-Th treatment in SK-OV3 and MDA-MB-231 tumors [26]. Copyright©2018, Springer Nature

Owing to the short half-life of free thrombin in the blood circulation and its inherent capacity to spontaneously trigger blood clotting when administered systemically, the high risk to the heart and brain resulting from thrombin cannot be ignored. To achieve the precise delivery of thrombin to tumor sites and trigger intratumoral formation of thrombi, Zhao’s group designed a DNA origami-based nanorobot to transport thrombin and present it specifically in tumors [26]. By attaching a DNA aptamer that can bind to nucleolin, the nanorobots were endowed with tumor-targeting capabilities by recognizing nucleolin receptors that are specifically expressed on the surface of tumor endothelial cells. The nanorobot responded to nucleolin and a conformational change from a closed state to an open state could be achieved, thus exposing internal thrombin and inducing the formation of a thrombus (Fig. 2a). The robot can not only protect thrombin from preleakage but also specifically transport thrombin into tumor blood vessels to achieve selective occlusion of tumor blood vessels (Fig. 2b, c). The results of the in vitro and in vivo stability studies showed that the DNA nanorobots could well maintain structural stability and thrombin activity under the experimental conditions. After intravenous injection (*i.v.*), the nanorobots efficiently targeted tumor tissue (Fig. 2d) and induced the *in-situ* formation of thrombi (Fig. 2e). The use of DNA nanorobots to deliver thrombin in vivo showed significant therapeutic effects in various tumors, such as breast cancer, melanoma, ovarian cancer, and primary lung cancer (Fig. 2f, g). Although the production costs of DNA are relatively high and there is a certain degree of difficulty in the construction of suitable DNA for tumor therapy, the successful application of thrombin in tumor embolization in this system is of great pioneering significance.

**Fig. 3:**
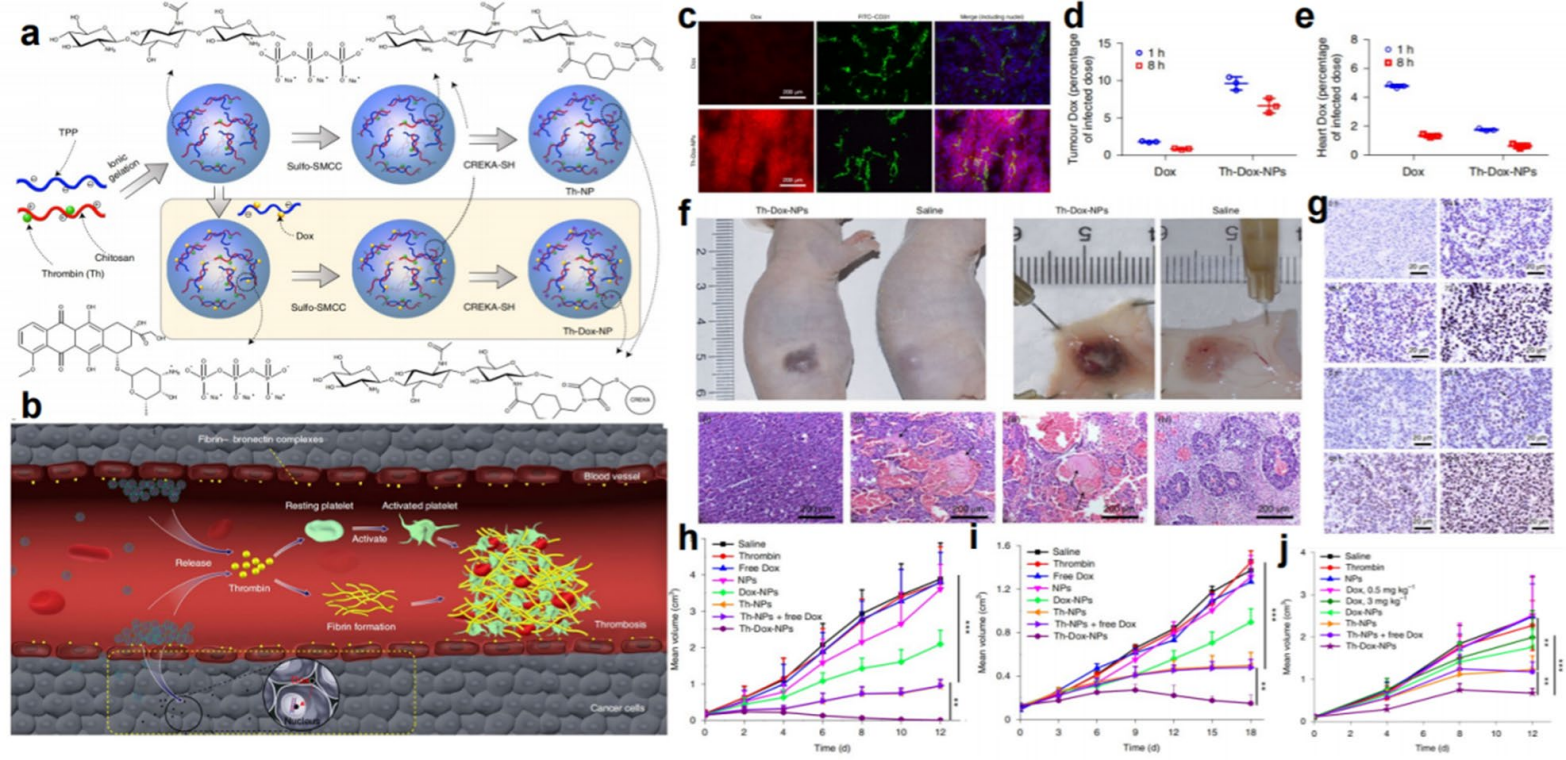
Combination of tumor embolization therapy and chemotherapy based on tumor-targeting nanoparticles. **a** The fabrication of chitosan-based polymer NPs and the loading of thrombin and Dox. **b** Schematic illustration of Th-Dox-NP activity in tumor tissue. **c** Confocal microscopy images of tumor sections from mice treated with free Dox or Th-Dox-NPs for 8 h. **d**, **e** The accumulation of Dox in tumor tissue and the heart. **f** Vascular disruption and thrombosis formation in tumors treated with Th-Dox-NPs. **g** Apoptosis in tumors treated with Th-NPs or Th-Dox-NPs at different time points. **h**–**j** The therapeutic effect in mice bearing B16-F10 melanomas, MDA-MB-231 human breast xenograft tumors, and MHCC97H human liver tumor [27]. Copyright©2020, Springer Nature

The above DNA nanorobot for the targeted delivery of the clinical hemostatic drug thrombin into tumor vessels can achieve local generation of tumor infarction and necrosis and demonstrates great potential for tumor treatment. However, the relatively high production costs for the construction of the DNA nanorobot may impede the clinical translation of the strategy to a certain extent. Hence, the same group developed a more economical nanocarrier by choosing the polymeric macromolecule chitosan to facilitate vascular-occlusion therapy [27]. Dox and human thrombin (Th) were coencapsulated into chitosan-based polymeric nanoparticles (NPs) with high load capacity (Fig. 3a). The CREKA peptide, which can specifically recognize tumor-overexpressed fibrin-fibronectin complexes, was conjugated to the surface of the nanoparticles to endow them with active tumor tissue-targeting ability (Fig. 3b). Compared with the nontargeted particles, the CREKA-conjugated NPs showed considerably higher tumoral accumulation and decreased heart exposure to Dox (Fig. 3c–e). Based on that, the formation of thrombi revealed by the fibrin-containing clots presented in the hematoxylin-and-eosin (H&E) staining images was clearly observed in the tumor vessels within 24 h (Fig. 3f). The Th-Dox-NPs developed in this work could kill tumor cells by two distinct aspects including cutting off the blood supply by thrombus and inhibiting tumor cell proliferation by Dox, therefore, the in vivo combination of embolization therapy and chemotherapy based on the Th-Dox-NPs exhibited an improved median survival (> 45.0 days) and highest tumor inhibition efficiency (80%) compared with single therapy-treated tumors (Fig. 3g–j). The synergistic effect was also achieved in rabbit models without obvious side effects. This strategy not only solves the safety problem of thrombin but also realizes the synergistic treatment of tumor embolization therapy and chemotherapy. Considering that the materials used in the fabrication of the nanoparticles are all clinically proven or biodegradable and the advantage of the approach in combining chemotherapeutic drugs with vascular infarction, this nanotherapeutic strategy holds great clinical translation potentials.

**Fig. 4:**
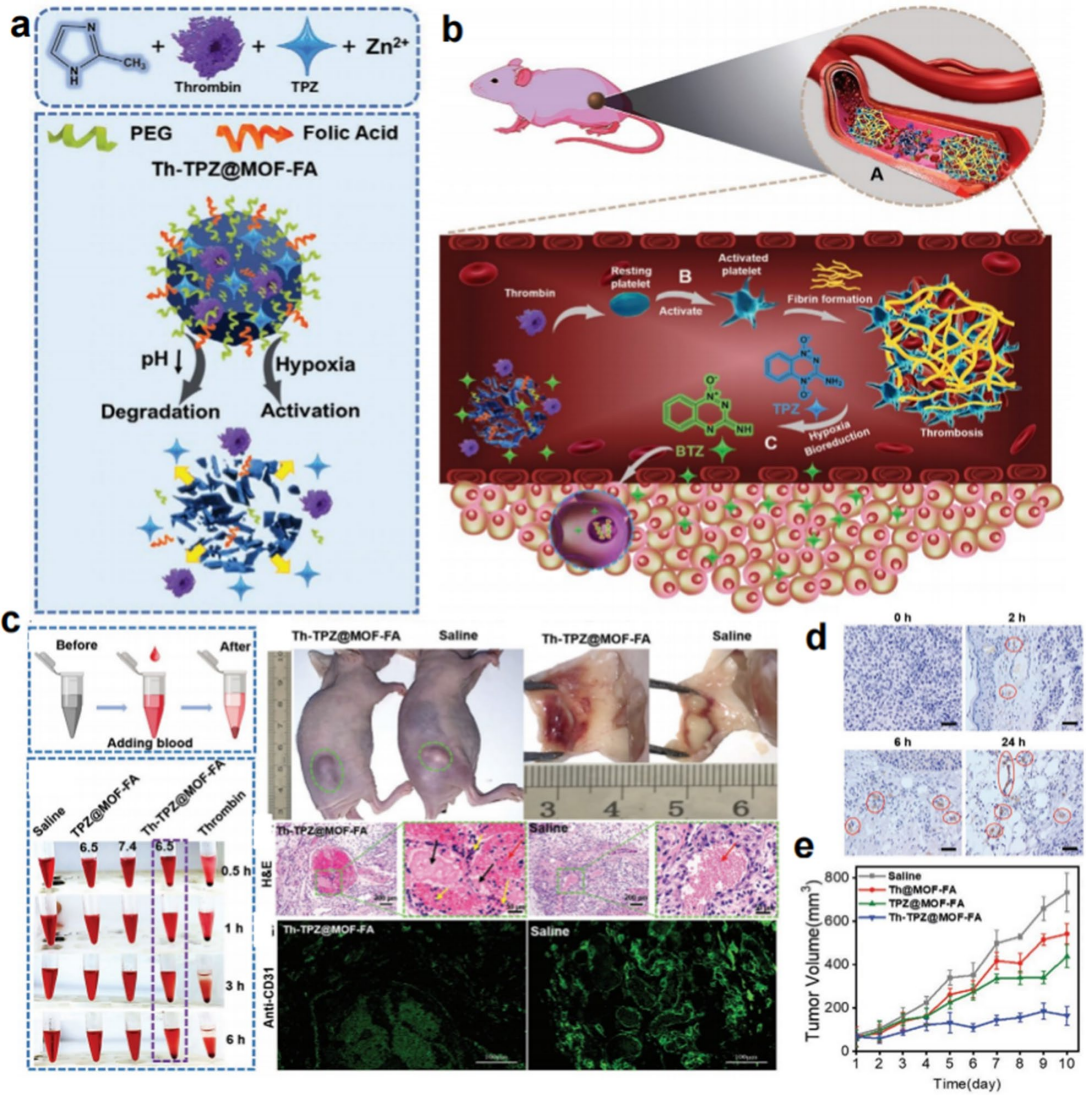
Thrombosis in the tumor triggers enhanced hypoxia-activated prodrug therapy. **a** Schematic fabrication of Th-TPZ@MOF-FA and the mechanism of degradation and prodrug activation. **b** Schematic illustration of the formation of thrombosis in the tumor and enhanced hypoxia-activated prodrug therapy. **c** Blood clot formation in vitro and thrombosis in tumors treated with  intravenous injection of Th-TPZ@MOF-FA. **d** CD 41-stained tumor sections at different time points after the administration of Th-TPZ@MOF-FA. **e** The in vivo antitumor activity of Th-TPZ@MOF-FA [32]. Copyright©2021 Wiley-VCH GmbH

The main mechanism of tumor blood vessel embolization in inhibiting tumor neovascularization and inducing tumor cell apoptosis is breaking up the supply of tumor nutrients and oxygen. The deprivation of oxygen would make the tumor more hypoxic, which can be an attractive target for tumor targeted therapy [28–31]. Hence, Ma et al. developed metal-organic framework (MOF) nanoparticles with the capacity of active tumor targeting to coencapsulate coagulation-inducing protease Th and a hypoxia-activated prodrug (HAP) tirapazamine (TPZ) [32]. Owing to the confined encapsulation properties of MOF and the mild synthesis conditions, the enzymatic properties of Th could be efficiently maintained, the catalytic active sites could be dispersed, and premature leaching of the contents could be remarkably prevented (Fig. 4a). After intravenous injection, the nanoparticles could be able to accumulate at the tumor site *via* the active targeting effect of folic acid (FA). Under the acidic tumor microenvironment (TME), Th-TPZ@MOF-FA was quickly degraded, resulting in the release of Th and TPZ. The spontaneously activated platelets and induced vascular infarction by Th further cut off the oxygen supply to significantly increase the level of hypoxia in the tumor site, thus triggering the bioreduction of TPZ to generate the toxic free radical BTZ for tumor cells killing (Fig. 4b). The ex vivo and in vivo blood clot formation shown in Fig. 4c indicated that the acid-activated Th-TPZ@MOF-FA successfully triggered the formation of thrombi. Further in vivo exploration of the stage of thrombus formation in the tumor site indicated that an advanced thrombus appeared in the tumor site after injection for 6 h, and dense thrombi could be observed at 24 h (Fig. 4d). Based on that, in vivo combination therapy was carried out, and the Th-TPZ@MOF-FA group demonstrated significant tumor suppression and obvious nuclear shrinkage and damage (Fig. 4e). This strategy solves the safety problem of Th, makes up for the insufficiency of single tumor embolization, and overcomes the limitation of inadequate hypoxia in hypoxia-activated prodrug treatment, further promoting the application of tumor embolization therapy combined with other conventional therapy methods.

### US-responsive nanoparticles

Owing to its high safety, noninvasiveness, deep tissue penetration, real-time visualization ability, and relative ease of access, ultrasound (US) has been widely used as an imaging modality worldwide. In addition to being used in diagnostic imaging, ultrasound is often used in the treatment of malignant tumors [33–35]. In particular, simultaneous real-time imaging and responsive drug release can be achieved when US is combined with an appropriate drug delivery system, resulting in precise on-demand drug delivery in organs and sites [36, 37]. For instance, ultrasound-responsive microbubbles can be destroyed through acoustic power specifically at the irradiated site, thus achieving drug/gene targeted delivery [33].

**Fig. 5:**
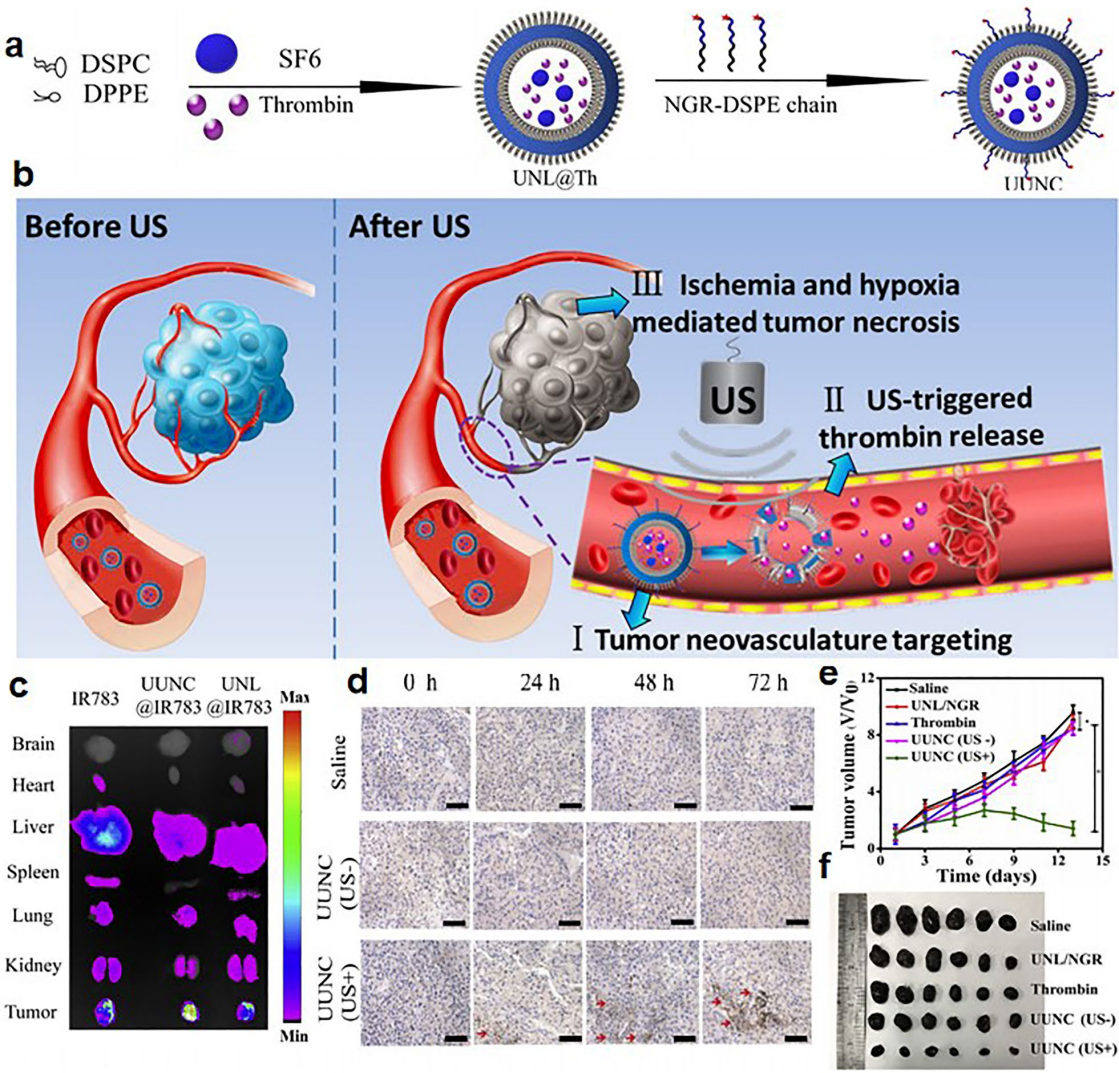
Ultrasound (US)-responsive nanoparticles with active targeting effects for tumor embolization therapy. **a** The fabrication of UUNC. **b** Schematic illustration of the mechanism of UUNC in tumor neovasculature targeting and US-responsive thrombus formation at the tumor site. **c** The tumor targeting ability of UUNC in vivo. **d** US-induced in situ thrombogenesis of UUNC within tumors detected by CD41 immunostaining assays. **e**, **f** The inhibition of tumor growth in different groups during treatment [38]. Copyright©2019 Elsevier B.V

Inspired by the application of US in tumor therapy, Shao et al. developed a US-responsive ultrasensitive nano “thrombus constructor” (UUNC) through the membrane hydration-mechanical vibration method for tumor non-interventional embolization therapy (Fig. 5a) [38]. Thr was loaded within the nanoliposome with a loading content of approximately 6.7 wt%, and SF6 gas was subsequently injected to endow it with US responsiveness (Fig. 5b). Meanwhile, NGR peptides, which exhibit an active targeting effect to tumor neovascularization, were attached to the nanoparticles. Taking advantage of the targeting ability to the tumor vessel, UUNC exhibited significantly higher tumor accumulation than the other groups (Fig. 5c). Under US treatment (1 W/cm^2^, 1 min), mice injected with UUNC resulted in more obvious thrombi in the tumor blood vessels, and the area of the formed thrombus increased and became dense with time than that in the mice treated with saline or UUNC (without US) (Fig. 5d). The US-triggered formation of thrombus based on UUNC induced a decrease in blood supply and deprivation of nutrients in tumors; therefore, significant tumor growth inhibition in the UUNC (with US)-treated group was achieved (Fig. 5e, f). Furthermore, due to the US-responsive properties and the tumor vascular-specific targeting effect, there were no noticeable abnormalities or thrombosis in major organs, demonstrating the high safety of UUNC. This work realized tumor blood vessel infarction and targeted tumor treatment with negligible toxicity *via* the combination of exogenous stimuli and internal active targeting ability.

### Laser irradiation-responsive nanoparticles

Stimulus-responsive materials have gained much attention during the past few decades due to their stability and flexibility. Among the commonly used endogenous pathological stimuli and external physical stimuli, laser irradiation has attracted great attention due to its high sensitivity, spatiotemporal controllability and easy modulation [21, 39–41].

**Fig. 6:**
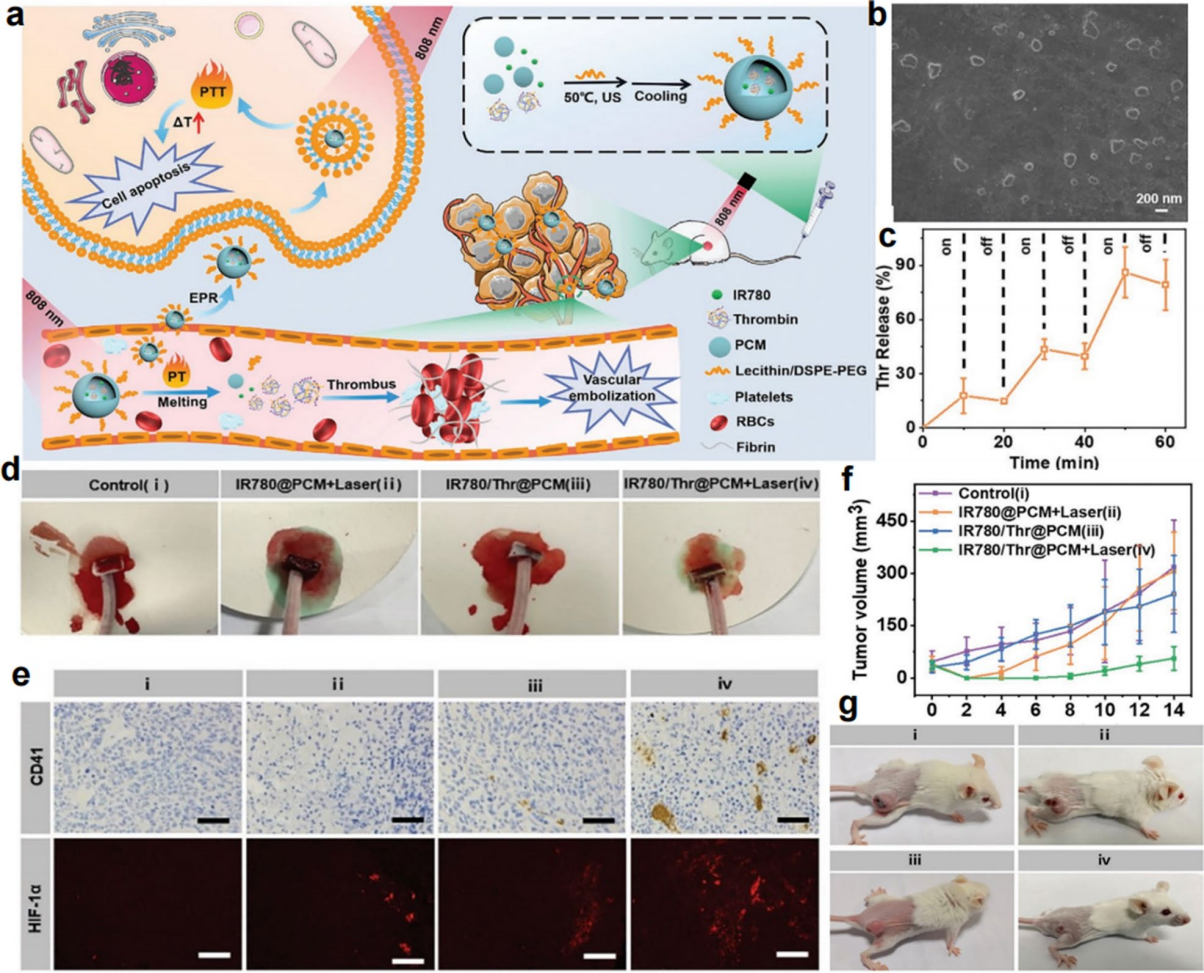
Tumor-specific embolization therapy based on photothermal-responsive nanoplatform. **a** Schematic illustration of the fabrication of IR780/Thr@PCM and its activity in the tumor site. **b** SEM image of IR780/Thr@PCM. **c** Laser irradiation triggered thrombin release. **d** The hemostatic efficiency of IR780/Thr@PCM in the mouse tail amputation model. **e** The detection of thrombi and the hypoxia level in the tumor. **f**, **g** Tumor growth in different groups [45]. Copyright©2021 Wiley-VCH GmbH

Phase change materials (PCMs) refer to those materials that have large latent heats of fusion and revisable transitions between the solid and liquid states. Owing to their tunable melting point, relatively low cost, high chemical stability, and good biocompatibility, PCMs have been widely utilized as thermoresponsive materials in tumor therapy [42–44]. Taking advantage of the wax seal property of PCMs, the payloads encapsulated within the PCMs can avoid preleakage and achieve responsive release under thermal control. Therefore, PCMs show great potential for utilization as Th carriers. The Dong group developed PCM-based nanocarriers for the safe delivery and controllable release of thrombin (Thr) by coencapsulating IR780 and Thr within PCM *via* a resolidification approach [45] (Fig. 6a). The fabricated IR780/Thr@PCM NPs exhibited a uniform distribution (Fig. 6b) and responsive drug release under laser irradiation (Fig. 6c). After intravenous injection, the IR780/Thr@PCM NPs exhibited a long blood circulation half-time and high tumor accumulation. Under 808-nm laser irradiation, the quick release of Thr in the tumor site could be observed due to the melting of PCMs triggered by the photothermal effect of IR780. The released Thr would further efficiently activate platelets and convert fibrinogen into fibrin to induce a strong coagulation reaction (Fig. 6c, d). The quickly formed thrombus in the tumor blood vessels broke down the supply of nutrition and oxygen for tumor cells, resulting in the serious apoptosis and necrosis of tumor cells (Fig. 6e–g). The encapsulation of PCMs protected Thr from being pro-released during blood circulation, thus improving the safety of Thr *via* intravenous injection. Meanwhile, the controlled release of the cargo achieved by light irradiation realized high specific generation of thrombi. Such a precise drug delivery system demonstrated promise for tumor embolization therapy and provided a high reference value for the delivery of substances that take effect in the blood for tumor therapy.

In another work, Zhang’s group fabricated thrombin-binding aptamer (TBA-Th) conjugates and conjugated them on Au NRs by thiol-terminated functional TBA to realize the loading of Th [46]. Meanwhile, tranexamic acid (TA), an antifibrinolytic agent, was introduced into the nanosystem by Cys-PEG8-IEGR-TA. The Th activity in blood circulation would be inhibited, while under laser irradiation, the photothermal effect generated by the Au nanorods (Au NRs) could trigger the release of Th and TA, resulting in the activation of the tumoral intravascular coagulation reaction and inhibition of the fibrinolysis process. By a photo-initiated cascade reaction, enhanced blood clots would be formed in the blood vessels to stably block the tumor blood vessels, and as a result, the metabolism of the tumor will be affected, thereby inhibiting the growth of the tumor.

The utilization of the thermal effect to trigger the release of Th in the tumor site exhibits good controllability and high efficiency. Considering that the activity of biological enzymes is usually related to the temperature at which they are located, it is necessary to reasonably control the temperature when using the thermal effect to trigger drug release, thus preventing the decreased activity of biological enzymes at high temperatures.

**Fig. 7:**
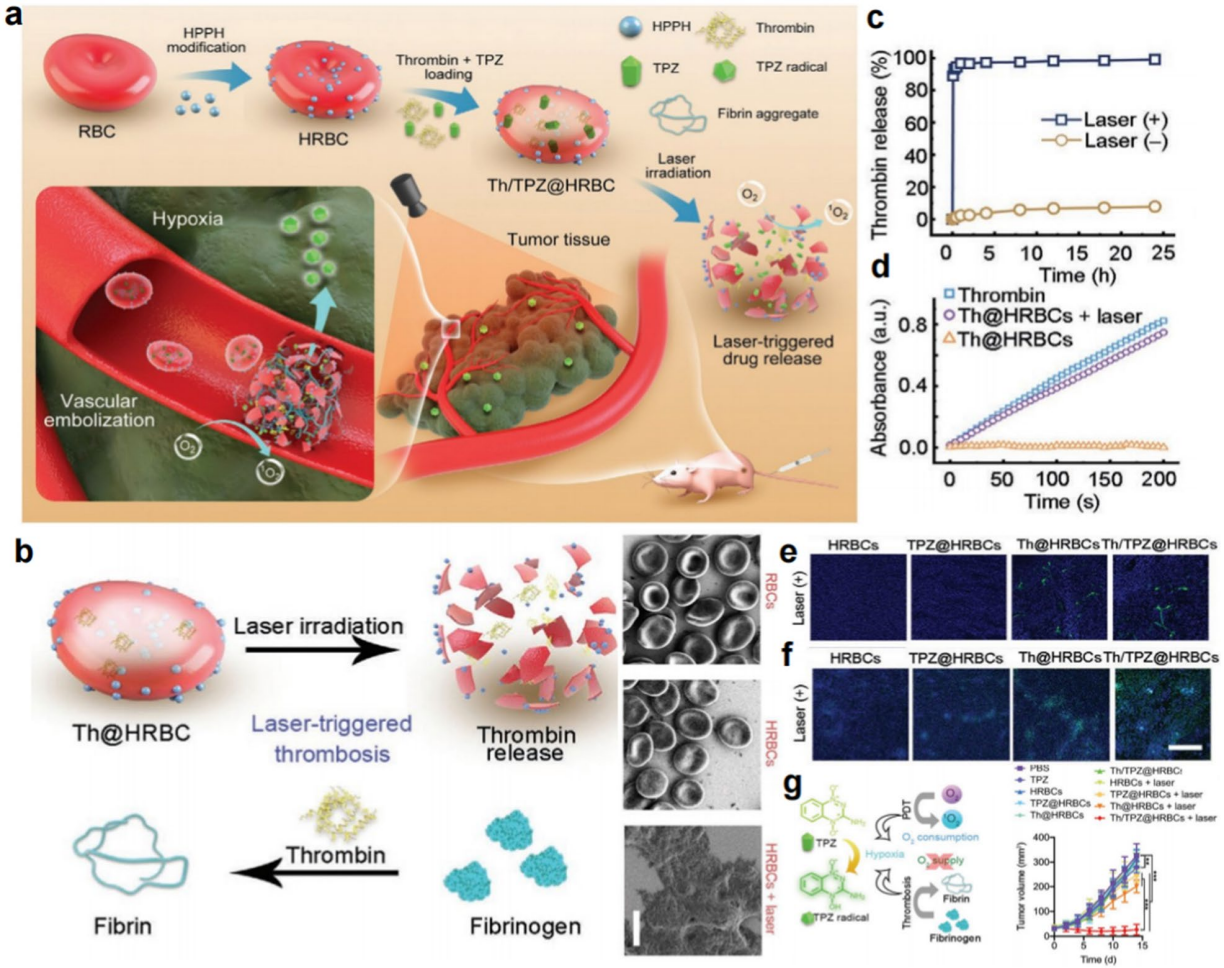
Photoactivatable bomb for tumor embolization therapy and hypoxia-activated chemotherapy. **a** The fabrication process and work mechanism of Th/TPZ@HRBCs. **b** Laser irradiation-triggered explosion of Th/TPZ@HRBCs. **c**, **d** The responsive release of thrombin with laser irradiation. **e** Ex vivo immunofluorescence staining assays of the tumor slices to indicate the formation of thrombi. **f** The hypoxia level in different groups. **g** The tumor inhibition efficiency of Th/TPZ@HRBCs [50]. Copyright©2021 Wiley-VCH GmbH

Red blood cells (RBCs) are the most abundant and longest-lived blood cells in the blood. Zhang’s group successfully developed the technology of biomimetic encapsulation of nanoparticles by wrapping the membrane protein on the surface of PLGA nanoparticles *via* the method of porous membrane extrusion for the first time in 2011 [47]. Since then, erythrocyte membrane-coated nanocarriers have opened up a new category of building cell-like drug delivery systems and have shown great potential in the applications of tumor diagnosis and treatment owing to their long-term blood circulation, excellent biocompatibility, and low immunogenicity [47–49]. Zhu et al. chemically anchored 2-(1-hexyloxyethyl)-2-devinyl pyropheophorbide-α (HPPH) to the surface of RBCs. The oxidative ^1^O_2_ generated by the obtained HRBCs under short and mild laser irradiation could trigger the fast release of the intracellular contents (Fig. 7a). Hence, they further encapsulated Th and TPZ inside HRBCs through hypotonic/hypertonic treatments (Th/TPZ@HRBCs) [50]. With 671-nm laser irradiation treatment for a short time (20 mW/cm^2^, 60 s), the Th/TPZ@HRBCs exploded (Fig. 7b), and nearly 90% of the Th was released (Fig. 7c, d). After intravenous injection, the burst release of Th induced by external laser irradiation could form local thrombi in tumor vessels (Fig. 7e), which might not only trap more circulating Th/TPZ@HRBCs in tumor regions but also deplete intratumoral oxygen and intensify the tumor hypoxia level (Fig. 7f). Therefore, the chemotherapeutic effect of TPZ was activated to inhibit tumor growth (Fig. 7g). Different from the traditional nano drug delivery systems that are easily cleared by the immune system or escape from vessels *via* the enhanced permeability and retention (EPR) effect, the HRBCs developed in this work showed an exciting effect in the targeted delivery of antitumor drugs, and proposed a novel strategy for therapeutic agents that take effect only in blood vessels.

With further research, different types of drug delivery systems (DDSs) based on organic or inorganic nanomaterials could be applied in loading substances with coagulant activity for tumor embolization therapy. Four main factors should be considered in the design of these DDSs: (1) The desirable nano DDSs should have appropriate diameter, morphology and surface modification; therefore, high drug loading efficacy, long blood circulation and efficient drug accumulation in the tumor site after intravenous injection could be achieved. (2) The designed nano DDSs should have a good protective effect on the loaded drug, preventing its premature release during the blood circulation, and can achieve responsive and rapid drug release at the tumor site. (3) Multifunctional NPs can be constructed by adding imaging contrast agents to visualize the therapeutic procedure. (4) The nano DDSs themselves should have low biotoxicity.

## Tumor microenvironment-responsive nanoparticles for embolization therapy

As the environment for tumor cells to survive, the TME is composed of immune cells, inflammatory cells, tumor-associated fibroblasts, microvessels, and various cytokines and chemokines around the tumor cells. Compared with normal tissues, the tumor microenvironment is characterized by abnormal blood vessels, hypoxia, acidity, high content of reactive oxygen species and reducing substances, immunosuppression, autophagy and metabolic changes [51–53]. These features provide different targets for the development of TME stimuli-responsive nanoparticles [39, 54, 55]. In tumor embolization therapy, stimuli-responsive nanoparticles are in an “invisible” state during circulation in vivo. Once they enter the tumor microenvironment, the special microenvironment will stimulate transitions such as phase or aggregate state transitions, thus achieving blood vessel obstruction. The environmental response of the nanoparticles would cause rapid enrichment at the tumor site and induce tumor embolization therapy. Therefore, TME-responsive nanomaterials show excellent potential as embolization agents.

### Copper- and phosphate-responsive nanoparticles

Due to the importance and unique characteristics of the tumor vascular system, tumor vascular targeting therapy, which always involves two main strategies, disrupting the angiogenesis pathway to prevent new blood vessel formation and obstructing or destroying established blood vessels in solid tumors, has gradually become a research hotspot [56–59]. Tumor blood vessels are not independent, and the new and existing blood vessels can take effect cooperatively to reduce the effect of vascular targeted drugs relying on a single working mechanism.

**Fig. 8:**
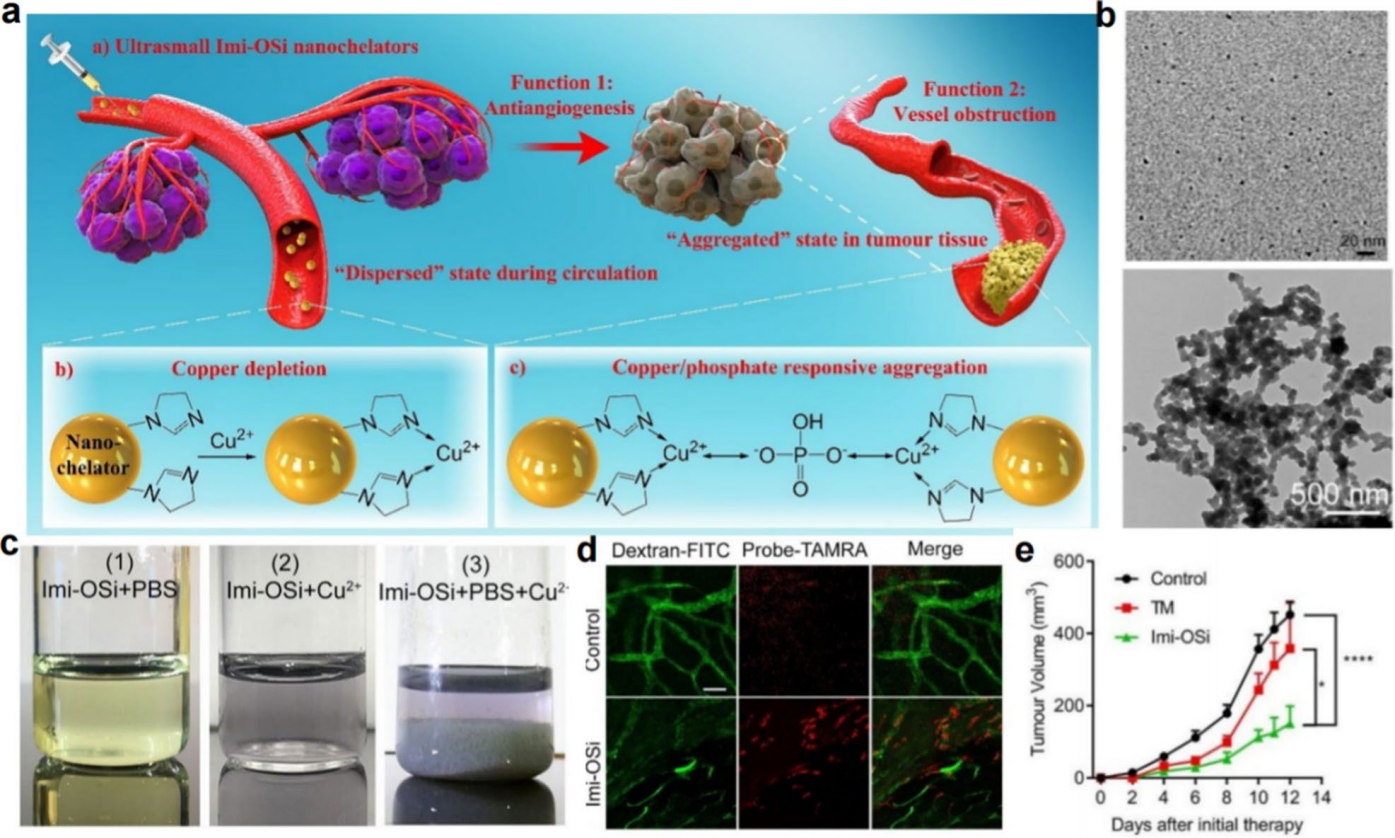
Responsively aggregatable sub6 nm nanochelators for tumor vasculature therapy. **a** The mechanism of Imi-OSi nanochelators in tumor vasculature-targeted therapy. **b** TEM images of the Imi-OSi nanochelators and the formed aggregates. **c** The change of the Imi-OSi nanochelator when mixed with PBS, Cu^2+^ or both PBS and Cu^2+^. **d** Representative tumor vessel images indicated that copper deficiency induced thinner tumor capillaries and the formation of blood clots. **e** The anticancer activity of Imi-OSi nanochelators [60]. Copyright©2019 American Chemical Society

Hence, Yang et al. developed sub6 nm responsively aggregative nanochelators, which integrated antiangiogenesis and vascular obstruction, and high efficiency of renal clearance, achieving improved antitumor activity and enhanced biosafety [60] (Fig. 8a). These ultrasmall nanochelators (ImiOSi) were fabricated *via* a one-pot hydrothermal method by using N-(3-triethoxysilylpropyl)-4,5-dihydroimidazole (TEDI) as the organosilica precursor and sodium citrate as the alkaline catalyst. The well-dispersed Imi-OSi nanochelators exhibited higher selectivity for copper ions (269 mg/g) than for other biologically relevant metal ions. Interestingly, when the Imi-OSi suspension was sequentially added to phosphate-buffered saline (PBS) and Cu^2+^, obvious aggregation was observed, indicating the formation of microsized aggregates (Fig. 8b, c). The chelation of Cu^2+^ by Imi-OSi would cause the depletion of bioavailable copper in the tumor site, which plays an important role in the secretion of multiple angiogenic factors for antiangeogenesis, thus resulting in antiangeogenesis. On the other hand, the thinner tumor capillaries caused by the depletion of Cu^2+^ would be further obstructed due to the formation of microsized aggregates (Fig. 8d). A remarkable tumor inhibition effect was found during the therapeutic period in both 4T1 breast tumors and CT26 colon tumors, demonstrating that Imi-OSi nanochelators developed in this work can inhibit different kinds of tumors, even tumors less sensitive to copper depletion, by integrating multiple antitumor mechanisms (Fig. 8e). Furthermore, due to the rapid renal clearance after intravenous injection, the Imi-OSi showed enhanced biosafety. This work for the first-time combined tumor vascular anti-angiogenesis and obstructing functions within one nanoparticle, offering great opportunities for tumor vasculature-targeted therapy.

### GSH/H_2_O_2_ responsive nanoparticles

The higher intracellular glutathione (GSH) or hydrogen peroxide (H_2_O_2_) levels in the TME than in normal tissue have become a fascinating target in the design of functional NPs for tumor therapy [61–64]. Among these GSH/H_2_O_2_-responsive NPs, MnO_2_-based NPs exhibited great potential for utilization as drug carriers due to their decomposition to Mn^2+^ after endocytosis [65–67].

**Fig. 9:**
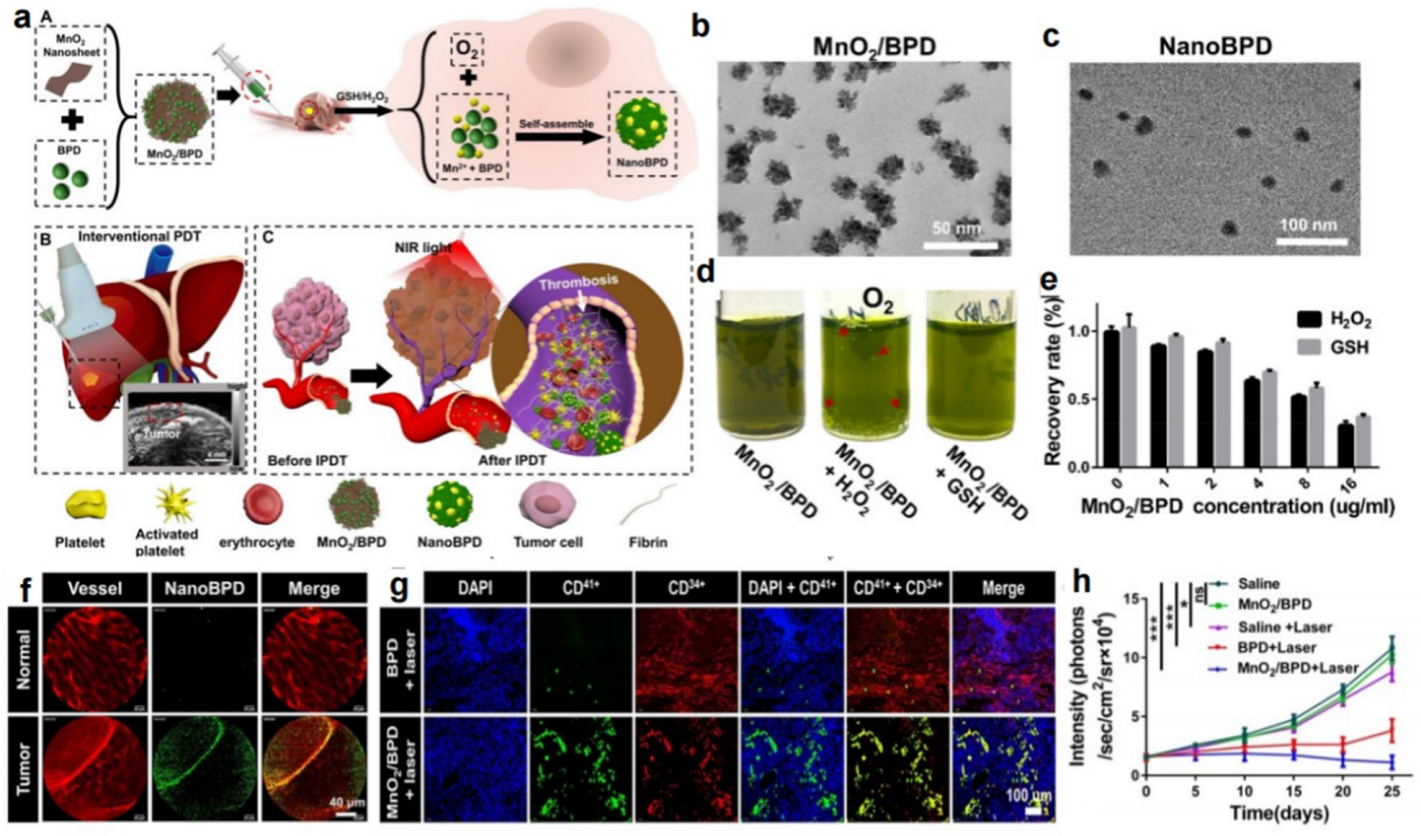
GSH/H_2_O_2_ responsive nanoparticles for tumor embolization therapy. **a** The MnO_2_/BPD fabrication and thrombosis formation induced by IPDT. **b**, **c** TEM images of MnO_2_/BPD and nanoBPD. **d** The generation of O_2_ and reduction of MnO_2_/BPD under different conditions. **e** The consumption of GSH and H_2_O_2_ in HUVECs by MnO_2_/BPD. **f** The specific targeting of tumor vessels mediated by MnO_2_/BPD. **g** Immunostained tumor slices to confirm the occluded blood vessels. **h** The antitumor effects in the HCC xenograft model [68]. Copyright©2020 American Chemical Society

The MnO_2_ nanosheets were prepared by Wang et al. and utilized as the carrier for verteporfin (a benzoporphyrin derivative [BPD]) (MnO_2_/BPD) [68]. The fabricated MnO_2_ nanosheets had a large surface area and a large amount of Mn–N coordinate bonding, resulting in the high loading efficiency of the photosensitizer BPD (93.67%). Meanwhile, BPD showed the ability to bind to the low-density lipoprotein receptor; thus, the MnO_2_/BPD were able to target tumor vascular endothelial cells (TVECs) (Fig. 9a). Under the conditions of high levels of intracellular GSH and H_2_O_2_, MnO_2_/BPD could be quickly reduced to a large amount of Mn^2+^ and BPD, resulting in the generation of oxygen and depletion of GSH (Fig. 9d, e). The nanoBPD could be further formed by the released Mn^2+^ and BDP *via* the reaction of Mn^2+^ with the porphyrin ring and carboxylate radicals in BPD (Fig. 9b, c). Under laser irradiation, the formed nanoBPD exhibited higher photodynamic therapy (PDT) efficiency than free BPD owing to the aggregation of free BPD in one nanoparticle. After intravenous injection, MnO_2_/BPD exhibited a remarkable tumor vascular targeting effect (Fig. 9f) and vessel density prediction, as revealed by in vivo magnetic resonance imaging (MRI), ultrasonic imaging (UI) and fluorescence imaging (FL). Based on that, they performed intervention PDT (IPDT) 24 h after intravenous injection of MnO_2_/BPD, and the MnO_2_/BPD + laser treated tumors exhibited obviously occluded blood vessels compared with the other groups (Fig. 9g). Meanwhile, the trimodal imaging methods were utilized to predict the tumor embolization efficacy since the imaging intensity and tumor volume change showed a negative correlation. Since the enhanced PDT could be able to kill TVECs to amplify the effect of PDT by inducing the coagulation cascade, the in vivo tumor therapy results showed that the MnO_2_/BPD + laser treated groups had the best therapeutic effect including tumor volumes and survival rate (Fig. 9h). This work not only provided novel nanoparticles for TME-triggered PDT and amplified the tumor embolization effect by the coagulation cascade but also proposed a desirable predictor to identify the therapeutic effect. In future research on GSH/H_2_O_2_-responsive nanoparticles for tumor embolization, it is desirable to develop nanoparticles with improved sensitivity to GSH/H_2_O_2_ since the level of GSH/H_2_O_2_ is always different in different kinds of tumors.

### Acidity responsive nanoparticles

**Fig. 10:**
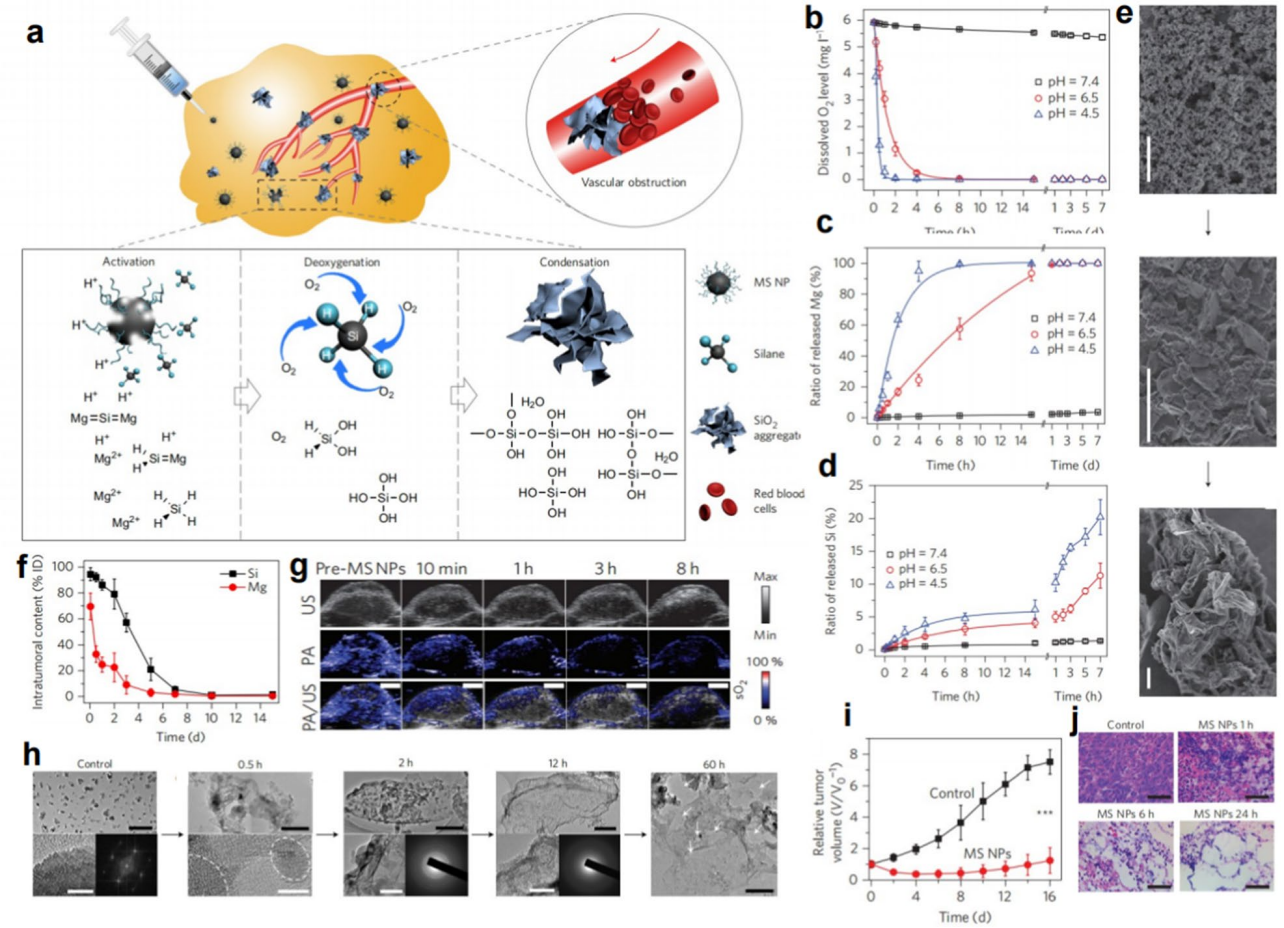
A deoxygenation agent based on magnesium silicide nanoparticles for tumor starvation therapy. **a** The mechanism of MS NPs for tumor-starving therapy. **b** Ohxygen consumption by MS NPs over time. **c**, **d** The release behaviors of Mg and Si. **e** The SEM images presented the transformation of MS NPs in morphology and size. **f** The intratumoral content of Mg and Si at different time points after the *i.t.* injection. **g** The time-course intratumoral sO_2_ levels indicated by PA images. **h** TEM images of MS NPs in the process of in vivo deoxygenation. **i**, **j** Antitumor effect of MS NPs [77]. Copyright©2017, Springer Nature

Tumor starvation therapy, which induces cell death by cutting off the blood supply that delivers oxygen and nutrients to the tumor, has become a promising therapeutic strategy [69–71]. On the other hand, direct removal of intratumoral oxygen would result in the inhibition of tumor growth, since oxygen is critical to the survival of tumors [72, 73]. The various deoxidants have some limitations in their utilization in tumor therapy, including poor biocompatibility, low deoxygenation efficiency, short-term oxygen scavenging, low tumor-tissue specificity and uninjectability [74–76]. Considering the above limitations, Shi’s group developed an injectable deoxygenating agent (DOA) with tissue penetration based on polyvinyl pyrrolidone (PVP)-modified Mg_2_Si nanoparticles (MS NPs) *via* the approach of self-propagating high-temperature synthesis (SHS) [77] (Fig. 10a). In an acidic TME, the Lewis base Si^4−^ ion in Mg_2_Si would enable silane release and initiate irreversible O_2_ consumption (Fig. 10b–d). During this process, not only the free oxygen but also the oxygen bound to hemoglobin could be completely scavenged, thus inducing serious hypoxia in the tumor and inhibiting the growth of tumor cells. On the other hand, the morphology of MS NPs would transform from a well-defined nanosheet into larger SiO_2_ microsheets not only in acidic media but also in tumor tissues (Fig. 10e–h), resulting in the blockage of the blood circulation system and the prevention of reoxygenation before degradation (Fig. 10f). After the intratumoral (*i.t.*) administration, MS NPs demonstrated desirable oxygen scavenging (Fig. 10g), high efficiency in inhibiting tumor growth and no detectable toxicity in the main organ tissues (Fig. 10i, j). Although less-effective tumor inhibition was achieved *via *intravenous injection in the context of clinical translation, the developed nanoparticles not only overcome the limitation of other deoxygenating agents but also show great promise for clinical tumor-starvation therapy with further performance improvement of the NPs.

The “one-size-fits-all” embolic materials have drawn great attention in enhancing therapeutic effects due to minimized complications and combination with other tumor treatments, such as chemotherapy and phototherapy. Lu et al. developed versatile and convenient theranostic nanocomposites with acidic TME-responsive sol-gel translation. The perfluoropentane was loaded within mesoporous Fe_3_O_4_, which was anchored with acidic microenvironment responsive poly [(L-glutamic acid-ran-L-tyrosine)-b-L-threonine-b-L-cysteine]s (PGTTCs). Owing to the existence of perfluoropentane (PFP) and m-Fe_3_O_4_, as well as the ability of the nanocomposites to label with ^131^I, the finally obtained PFP-m-Fe_3_O_4_@PGTTCs exhibited good effects in MRI, UI and single-photon emission computed tomography (SPECT) imaging. By adding an alternating magnetic field (AMF) (7 kW, 80% output, 375 kHz), the magnetic hyperthermia effect generated by m-Fe_3_O_4_ could be achieved in tumor therapy. Meanwhile, the gel formed by the good sol-gel transition properties of PFP-m-Fe_3_O_4_@PGTTCs in acidic TME could be able to embolize blood vessels, thus achieving non-interventional target-embolization therapy [78]. Due to the high tumor accumulation and specific embolization effects, tumor growth could be remarkably inhibited in both the H22-tumor-bearing mouse model and the VX2-tumor-bearing rabbit model. This pH-responsive embolization theranostic system not only overcame the shortcomings in TAE but also provided a novel theranostic candidate for combination embolization with other tumor therapies.

Calcium carbonate nanoparticles (CaCO_3_ NPs), a typical acid-responsive inorganic salt, have been proven by the Food and Drug Administration (FDA) in clinical applications. Ionized calcium (Ca^2+^) can activate the transformation of prothrombin, thus inducing blood coagulation. Based on that, Zhao’s group first explored the blood coagulation effect of CaCO_3_ NPs by acid stimulus both in vitro and in vivo [79]. At pH 5.0, CaCO_3_ NPs exhibited fast degradation and released a large amount of Ca^2+^, which could further play a role in inducing blood coagulation in different mimicking environments, including blood flow, TME and endosomes/lysosomes. When the tumor was intratumorally injected with CaCO_3_ NPs, thrombosis formation in the tumor vasculature was clearly observed. These results indicated that acid-responsive CaCO_3_ NPs have great potential in inducing tumor vasculature blockage to generate the tumor starving therapy.

In addition to the abovementioned materials, pH-responsive polymers can also be used as embolic materials [80, 81]. When an aqueous solution of a polymer embolic agent whose responsive pH value is similar to or the same as the pH of the acidic TME is injected into the tumor site, the polymer solution will change from sol to gel as the pH of the tumor tissue decreases, thereby blocking the tumor blood vessels and achieving the purpose of tumor growth inhibition. Based on that, Lu et al. developed PGTTCs-coated Au or Fe_3_O_4_ NPs for tumor noninterventional targeted embolization and thermal therapy [81]. The smart composites could change from sol to gel in an acidic microenvironment, thus occluding the blood vessels of tumors after intravenous injection to inhibit tumor growth to some extent. When combined with thermal therapy generated by laser irradiation (Au) or an alternative magnetic field (Fe_3_O_4_), the long-term survival rate (more than 80%) and significant therapeutic effects could be achieved within 15 days. The strategy developed in this work can effectively avoid the use of a microcatheter, thus minimizing the complications and risks and reducing the side effects. It also provides valuable guidance for the combination of tumor noninterventional embolization therapy and other therapeutic methods.

Given the detailed understanding of the difference between the TME and normal tissue, TME stimulus-responsive nanoparticles show great advantages in tumor embolization therapy, including enhancing therapeutic specificity and reducing side effects. To fulfill the roles of nanoparticles in tumor embolization therapy, endogenous stimuli-responsive nanoparticles should be designed to overcome the limitation of tumor heterogeneity. More endogenous stimulus factors, such as enzymes, hypoxia, or angiogenesis, can be exploited. Furthermore, considering that some of the stimuli may not be enough to trigger drug release or morphological transition of nanoparticles, the sensitivity of these nanoparticles should be improved.

## Peptide-based nanoparticles for tumor embolization therapy

As a new type of biomedical material, peptide polymers combine the abundant biological activity of peptide materials and the diversity of easy synthesis and response of polymers, which can further realize the design and synthesis of functional materials. Combining functional peptides such as targeting peptides, membrane-penetrating peptides, therapeutic peptides, and response peptides, with polymers can achieve higher specific selection and treatment efficiency for tumor. Therefore, polypeptide play an irreplaceable role in disease treatment, especially tumor treatment.

Truncated tissue factor (tTF) is composed of an extracellular region and a transmembrane region. The coagulation activity of the free extracellular region is very low since the coagulation function of tTF is mainly performed by the extracellular region, and the extracellular region must be fixed on the cell membrane to activate the coagulation function. To fulfil the function of tTF as a desirable procoagulant agent for tumor embolization therapy, tumor vessel targeting ligands should be introduced [82–84]. Zou et al. constructed the fusion protein tTF-EG3287 by a genetic engineering method. tTF is the recombinant form of tissue factor, which is the initiator of the extrinsic coagulation pathway [85]. EG3287 is a targeting polypeptide with the ability to specifically target Neuropihn-1 (NRP-1), which is a new target of tumor blood vessels. The fusion protein tTF-EG3287 could target tumor blood vessels by binding to highly expressed NRP-1, thereby anchoring tTF to the surface of the endothelial cell membrane. Membrane-bound tTF further restored the procoagulant activity of intact TF and then promoted the coagulation reaction in tumor blood vessels to form thrombi, which further caused thromboembolism to block the vascular supply of oxygen and nutrition for tumors. However, the affinity of EG3287 was still low, and the effect of tTF-EG3287 on inducing thrombi was not satisfactory in this work. Hence, the same group further constructed O-carboxymethyl chitin-coated Fe_3_O_4_ nanoparticles (OCMC/Fe_3_O_4_) and utilized them as drug carriers for tTF-EG3287 [86]. By adding a magnetic field to the tumor area, the obtained magnetic targeting procoagulant protein (MTPCP) could be quickly enriched in the tumor vessels and further bound to NRP-1 of TVEC by EG3287, resulting in the selective generation of coagulation in tumor-associated blood vessels. Tumor vascular embolization could inhibit tumor growth and cause vascular necrosis of tumor tissue. This study successfully constructed a new type of magnetic nanoprocoagulant protein, which is expected to be a safe, effective and convenient embolic agent for tumor vascular embolization therapy.

Synergistic therapy including two or more antitumor approaches has been reported to be an effective way to inhibit tumor growth. To utilize the advantages and overcome the drawbacks of each modality, Luo et al. synthesized virus-inspired gold nanorod-mesoporous silica core–shell nanoparticles integrated with tTF-EG3287 (GNR@VSNP-tTF-EG3287) for synergetic photothermal therapy (PTT) and selective vascular thrombosis therapy [87]. GNR@VSNP-tTF-EG3287 exhibited superior cellular uptake properties due to the unique topological viral structures, resulting in desirable antitumor efficacy. Meanwhile, the hyperthermia generated by GNR under laser irradiation could induce a high percentage of apoptosis of vascular endothelial cells, leading to a large number of phospholipid sites for tTF-EG3287 to exert its procoagulant activity. The combination of vascular blockage and PTT for in vivo tumor therapy provides a promising strategy for improving therapeutic effects by simultaneously inhibiting the tumor blood supply and tumor cell proliferation.

**Fig. 11:**
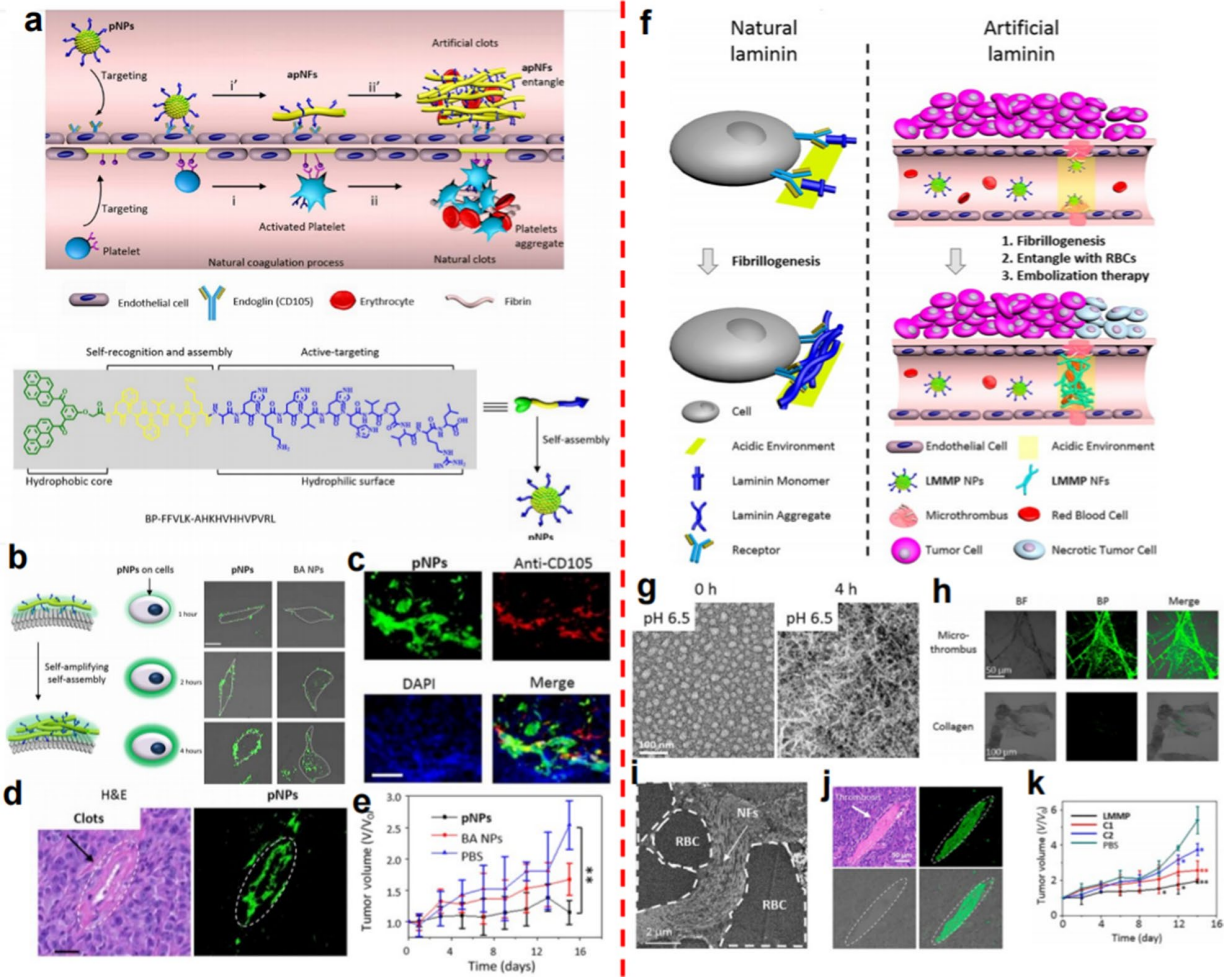
The mimic nanoparticles for precise embolization. **a** Platelet-mimicking nanoparticles for artificial coagulation. **b** The transformation of pNPs into apNFs. **c** The active targeting of pNPs to the tumor vessels. **d** The formation of artificial clots indicated by H&E staining and CLSM images. **e** Tumor growth was inhibited by pNP-induced tumor thrombosis [89]. **f** Comparison of fibrillogenesis in natural laminin and peptide-based nanoparticles mimicking laminin. **g** The transformation of LMMP morphology at pH 6.5. **h** The targeting effect of LMMP NPs when incubated with the microthrombus and collagen. **i** The entanglement of LMMP NFs and RBCs. **j** Vessel occlusion colocalized with LMMP NFs. **k** Tumor growth inhibition after intravenous injection with LMMP NPs [92]. Copyright©2020 American Chemical Society

Platelets, which play a critical role in coagulation formation, have attracted great attention [22, 88]. Inspired by the natural coagulation mechanism, platelet-like nanoparticles (pNPs) were designed by Wang et al. based on self-assembled peptides (BP-FFVLK-AHKHVHHVPVRL), achieving artificial coagulation to block tumor blood vessels (Fig. 11a) [89]. Bis-pyrenes (BPs), which were the hydrophobic core, exhibited an aggregation-induced emission (AIE) effect to enable the real-time observation of the in vivo behaviors of the nanoparticles. The FFVLK sequence, which was derived from amyloid peptide, could form fibrous structures. The AHKHVHHVPVRL sequence showed a specific targeting effect on the tumorally overexpressed transmembrane glycoprotein (endoglin, CD105) receptor in the activated endothelial cells (ECs), resulting in the transformation of pNPs (Fig. 11b). After intravenous injection, the activated platelet-like nanofibers (apNFs) were induced after the pNPs targeted and bound to the ECs (Fig. 11c). More binding sites could be further provided by the formed apNFs, thus the continuous activation and amplified self-assembly of pNP were triggered to induce the formation of artificial clots to block the tumor blood vessel (Fig. 11d). Based on that, the tumor inhibition rate was as high as 53% in the pNPs (2.4 mg/kg)-treated mice (Fig. 11e), much higher than that of clinically used drugs that target angiogenesis such as sunitinib and sorafenib. The strategy developed in this work showed great potential for not only tumor therapy but also dysfunctional vasculature diseases related to platelets.

The aggregation of laminin modulated by ligand–receptor interactions and decreased pH could be utilized as a basement membrane to efficiently prevent the movement of cells [90, 91]. Inspired by the property of laminin aggregates, the above group further designed laminin mimic peptide (LMMP)-based nanofibers for tumor growth inhibition [92] (Fig. 11f). In the LMMP, the Lys-LeuVal-Phe-Phe (KLVFF) sequence containing hydrogen bonding, targeting peptide Cys-Arg-Glu-Lys-Ala (CREKA) and pH-responsive sequence His6 were responsive to fibrillation, binding to the microthrombus in tumor vessels and modulating the fibrillation speed, respectively. BP is introduced to induce the formulation of the LMMP and endowed bright fluorescence with LMMP for in vivo observation. The LMMP NPs actively targeted the tumor blood vessels and transformed from the nanoformulation into nanofibers (NFs) with high specificity in the acidic TME (Fig. 11g, h). The laminin-like NFs attached to the microthrombus could further capture red blood cells in the bloodstream to form an *in-situ* embolus, resulting in higher tumor accumulation and longer-term retention for reduced administration frequency (Fig. 11i, j). The precise and fast-speed formation of blockage in the tumor blood vessels and the long-term blockage effect showed a significant effect in the inhibition of tumor growth with negligible toxicity (Fig. 11k).

Polypeptides have inherent bioactivity, biodegradability and biocompatibility, as well as adjustable structure and abundant functions, which exhibit broad prospects in tumor therapy. However, polypeptides often have high susceptibility to proteolysis, and it is necessary to modify the peptide to increase its in vivo stability to better apply it to tumor treatment.

## Conclusions and perspectives

Compared with traditional therapeutic strategies that target tumor cells, tumor embolization therapy exhibits unique advantages, such as the ability to efficiently induce a large amount of tumor cell death in a short period of time, no requirement for contact between the drug and tumor cells, and reduced drug resistance of tumor cells. With the development of nanotechnology, noninvasive embolization therapy based on the development of nanomaterials has shown the following advantages in overcoming the limitations of clinical transcatheter arterial embolization:

1. The size effect of nanomaterials makes them smaller in size and more easily exuded from abnormal endothelial cells so that they can be effectively retained in tumors over time. The development of new functional tumor non-interventional embolization materials based on nanomaterials can simplify the treatment process of TAE embolization, and through the functional expansion of nanoembolization materials, the transformation from single-functional biomaterials to multifunctional composite materials can be realized;
2. The nanodrug-loading system developed based on nanomaterials can achieve active tumor targeting through surface modification, which can not only achieve efficient loading of agents with procoagulant activity but also achieve targeted enrichment at the tumor sites and improve tumor embolization efficiency;
3. The development of stimuli-responsive nanocarriers further offers great potential for tumor embolization therapy. The use of stimuli-responsive nanocarriers can realize the stability of drugs in the blood circulation process and rapid spatiotemporal release at the tumor site. Meanwhile, specific embolization at tumor sites can be realized with reduced systemic toxicity by using the endogenous stimuli-responsive properties of nanoparticles.
4. The use of nanomaterials prepared from materials with good biosafety and biodegradability as embolization agents can solve the safety problems caused by traditional embolization agents.

With the development of nanomedicines, tumor embolization therapies with different mechanisms have been reported; and have achieved good therapeutic effects with reduced side effects. However, there are still some limitations of nanomedicines in the progress of clinical translation: (1) Substances with procoagulant activity, such as Th, have a strong coagulation function, but they do not have the ability to target tumor blood vessels. Although targeted delivery of Th can be achieved by nanocarriers, when Th is released, there is still a risk of ectopic embolism following blood flow to other blood vessels. (2) The nanoplatforms could accumulate in normal tissues, resulting in serious side effects. Therefore, it is urgent to develop eliminated nanoparticles that can be completely cleared. (3) More preclinical studies need to be carried out since the therapeutic effect of nanomedicines might not be consistent between the models of animal human patients.

Tumor non-interventional embolization therapy eliminates tumor cells in a unique way, showing great promise. With the boom in the development of nanotechnology, smart nanotherapeutics have offered unprecedented potential for non-interventional embolization therapy. By taking advantage of nanotechnology, it has gradually become a trend to develop targeted non-interventional embolization therapy strategies with good tumor targeting and  fewer toxic side effects. In future research, to achieve desirable non-interventional embolization therapy, nanoplatforms can be functionally designed based on the specific structural and functional characteristics of the tumor vasculature and modified with reference to the required medical environment to develop integrated medical embolization biomaterials with different functions or multiple functions. These new medical biomaterials are of great significance to the diagnosis and treatment of different types of tumors.

## Data Availability

Not applicable.

## Abbreviations

TAE: Transcatheter arterial embolization
i.v.: Intravenous injection
Th/Thr: Thrombin
NP: Nanoparticle
MOF: Metal-organic framework
HAP: Hypoxia-activated prodrug
TPZ: Tirapazamine
FA: Folic acid
TME: Tumor microenvironment
US: Ultrasound
PCMs: Phase change materials
TA: Tranexamic acid
RBCs: Red blood cells
HPPH: 2-(1-hexyloxyethyl)-2-devinyl pyropheophorbide-α
DDSs: Drug delivery systems
TEDI: *N*-(3-triethoxysilylpropyl)-4,5-dihydroimidazole
PBS: Phosphate-buffered saline
GSH: Glutathione
H_2_O_2_: Hydrogen peroxide
TVECs: Tumor vascular endothelial cells
IPDT: Intervention PDT
MRI: Magnetic resonance imaging
DOA: Deoxygenating agent
PVP: Polyvinyl pyrrolidone
SHS: Self-propagating high-temperature synthesis
SPECT: Single-photon emission computed tomography
AMF: Alternating magnetic field
FDA: Food and Drug Administration
NRP-1: Neuropihn-1
tTF: Truncated tissue factor
pNPs: Platelet-like nanoparticles
AIE: Aggregation-induced emission
ECs: Endothelial cells
apNFs: Activated platelet-like nanofibers
LMMP: Laminin mimic peptide
